# Anti-angiogenic and antioxidant effects of axitinib in human retinal endothelial cells: implications in diabetic retinopathy

**DOI:** 10.3389/fphar.2024.1415846

**Published:** 2024-06-17

**Authors:** Francesca Lazzara, Federica Conti, Pradip K. Sasmal, Shanavas Alikunju, Settimio Rossi, Filippo Drago, Chiara Bianca Maria Platania, Claudio Bucolo

**Affiliations:** ^1^ Department of Biomedical and Biotechnological Sciences, School of Medicine, University of Catania, Catania, Italy; ^2^ Dr. Reddy’s Laboratories Ltd., Hyderabad, India; ^3^ Eye Clinic, Multidisciplinary Department of Medical, Surgical and Dental Sciences, University of Campania “Luigi Vanvitelli”, Napoli, Italy; ^4^ Center for Research in Ocular Pharmacology-CERFO, University of Catania, Catania, Italy

**Keywords:** axitinib, retina, angiogenesis, diabetes, Nrf2/Keap1

## Abstract

Diabetic retinopathy is a secondary microvascular complication of diabetes mellitus. This disease progresses from two stages, non-proliferative and proliferative diabetic retinopathy, the latter characterized by retinal abnormal angiogenesis. Pharmacological management of retinal angiogenesis employs expensive and invasive intravitreal injections of biologic drugs (anti-vascular endothelial growth factor agents). To search small molecules able to act as anti-angiogenic agents, we focused our study on axitinib, which is a tyrosine kinase inhibitor and represents the second line treatment for renal cell carcinoma. Axitinib is an inhibitor of vascular endothelial growth factor receptors, and among the others tyrosine kinase inhibitors (sunitinib and sorafenib) is the most selective towards vascular endothelial growth factor receptors 1 and 2. Besides the well-known anti-angiogenic and immune-modulatory functions, we hereby explored the polypharmacological profile of axitinib, through a bioinformatic/molecular modeling approach and *in vitro* models of diabetic retinopathy. We showed the anti-angiogenic activity of axitinib in two different *in vitro* models of diabetic retinopathy, by challenging retinal endothelial cells with high glucose concentration (fluctuating and non-fluctuating). We found that axitinib, along with inhibition of vascular endothelial growth factor receptors 1 (1.82 ± 0.10; 0.54 ± 0.13, phosphorylated protein levels in fluctuating high glucose *vs* *.* axitinib 1 µM, respectively) and vascular endothelial growth factor receptors 2 (2.38 ± 0.21; 0.98 ± 0.20, phosphorylated protein levels in fluctuating high glucose *vs* *.* axitinib 1 µM, respectively), was able to significantly reduce (*p* < 0.05) the expression of Nrf2 (1.43 ± 0.04; 0.85 ± 0.01, protein levels in fluctuating high glucose *vs* *.* axitinib 1 µM, respectively) in retinal endothelial cells exposed to high glucose, through predicted Keap1 interaction and activation of melanocortin receptor 1. Furthermore, axitinib treatment significantly (*p* < 0.05) decreased reactive oxygen species production (0.90 ± 0.10; 0.44 ± 0.06, fluorescence units in high glucose *vs* *.* axitinib 1 µM, respectively) and inhibited ERK pathway (1.64 ± 0.09; 0.73 ± 0.06, phosphorylated protein levels in fluctuating high glucose *vs* *.* axitinib 1 µM, respectively) in HRECs exposed to high glucose. The obtained results about the emerging polypharmacological profile support the hypothesis that axitinib could be a valid candidate to handle diabetic retinopathy, with ancillary mechanisms of action.

## 1 Introduction

Diabetic retinopathy (DR) is a common complication of diabetes, which can lead, if not properly diagnosed and treated, to irreversible vision loss. Flaxman et al., 2017 estimated that in World Health Organization (WHO) European Region, including 53 countries, 950,000 individuals have visual impairment or blindness accountable to diabetic retinopathy (Flaxman et al., 2017). This estimation was confirmed for 2020, accounting to DR the 2.5% of cases of irreversible vision loss in people aged 50 years old or older (GBD 2019 Blindness and Vision Impairment Collaborators and Vision Loss Expert Group of the Global Burden of Disease Study, 2021). DR progresses from non-proliferative to proliferative stage, the latter characterized by abnormal retinal vessel growth, which causes macular edema, that if not treated can lead to retinal detachment and then irreversible vision loss. Intravitreal anti-vascular endothelial growth factor (anti-VEGF) agents (bevacizumab, ranibizumab, aflibercept) are the standard of care for treatment of diabetic macular edema (DME) (Hatamnejad et al., 2024), but recently also brolucizumab and faricimab have been approved for DME treatment (Montesel et al., 2021; Wong et al., 2023). In some cases, also intravitreal steroids, such as dexamethasone and triamcinolone acetonide are indicated to treat DME (Bucolo et al., 2015; Bucolo et al., 2018). Besides these mentioned pharmacological treatments, several unmet medical needs affect diabetic retinopathy management: burden of care due to high cost and invasive intravitreal treatments, poor adherence, and 40%–50% of cases refractory to current approved pharmacological interventions (Hatamnejad et al., 2024; Mandava et al., 2024). In this perspective in search of alternatives to intravitreal anti-VEGF agents, we focused our study on axitinib, a tyrosine kinase inhibitor (TKI).

Axitinib is currently approved for the treatment of advanced renal cell carcinoma (RCC), as second-line treatment in patients unresponsive to sunitinib (Bellesoeur et al., 2017). In particular, axitinib has lower IC_50_ towards VEGFR1-2-3 and PDGFRα-β, compared to sunitinib (Roskoski, 2007; Hu-Lowe et al., 2008), this difference is of about 2 log (150 fold). Both axitinib and sunitinib have been tested in non-clinical studies as anti-angiogenic agents for treatment of ocular neovascular diseases. In particular, axitinib exhibited protective effects in *in vitro* and *in vivo* models of retinal hypoxia (Kernt et al., 2012), age-related macular degeneration (AMD) (Thiele et al., 2013; Shi et al., 2019) and corneal neovascularization (Kang et al., 2013; Lledó Riquelme et al., 2018). However, sunitinib treatment has been associated with several ocular toxicity events and adverse effects, such as retinal detachment and exacerbation of retinal oxidative stress (Dib et al., 2012; Bayyoud et al., 2014; Fraunfelder and Fraunfelder, 2018; Santana-Garrido et al., 2020). On the contrary, axitinib has a good ocular safety profile and toxicity is less common, so it would represent a valid alternative to sunitinib as treatment of retinal neovascular diseases.

Advantage of use of a small molecule for treatment or adjuvant treatment of retinal diseases, compared to intravitreal anti-VEGF or corticosteroids, may arise from design of topical ocular formulations of this drug, and we already demonstrated that nanotechnological formulation of sorafenib (another TKI) for treatment of retinal angiogenesis, is a suitable pharmacological development strategy (Santonocito et al., 2021). Although the activity of axitinib as tyrosine kinase inhibitor is already well characterized (Choueiri, 2008; Kelly and Rixe, 2010; Escudier and Gore, 2011), we hereby explored the polypharmacological profile of axitinib, through a bioinformatic and molecular modeling approach, integrating these predictions with *in vitro* approaches. Basic and clinical research evidenced that in DR cell pathogenic mechanisms involve not only vessels but also neurons and glia (Platania et al., 2019; Amato et al., 2021). On this regard, retinal protection is an important issue in DR that it is commonly investigated both *in vitro* and *in vivo* (Bucolo et al., 2006; Wang et al., 2016). Besides *in vivo* models give important hints on pathogenic mechanisms and corroborate *in vitro* evidence of drug efficacy; we hereby used *in vitro* models of diabetic retinopathy, which are valuable tools for test antiangiogenic activity of drugs. Most common *in vitro* models of DR involve human retinal endothelial cells challenged with high glucose concentrations (Lazzara et al., 2022), but we hereby included, as alternative model, also fluctuating glucose concentrations.

Through an *in silico* target scouting approach and based on axitinib structure, we predicted that this compound would exert antioxidant activity by means of direct and indirect induction of Nrf2. We then explored *in silico* the activity of axitinib as Keap1 covalent ligand (Pittalà et al., 2017), and as putative melanocortin receptor ligand, known to be involved in regulation of angiogenesis and oxidative stress (Maisto et al., 2017; 2019; Gesualdo et al., 2021) in *in vitro* and *in vivo* models of diabetic retinopathy.

In the present study we investigated, in retinal endothelial cells challenged with high glucose concentrations (fluctuating and non-fluctuating), the anti-angiogenic effect of axitinib, evaluating VEGFR1 and VEGFR2 activation. Moreover, the main aim and novelty of our study was to investigate the polypharmacological profile, predicted *in silico*, of axitinib in two *in vitro* models of diabetic retinopathy, evaluating reactive oxygen species (ROS) production, ERK and Nrf2 protein activation.

## 2 Methods

### 2.1 Target scouting

Prediction of putative targets of axitinib was carried out accessing to SwissTargetPrediction webserver (http://www.swisstargetprediction.ch/ access: May 2021). Data were matched along with axitinib targets, that were retrieved from PubChem (https://pubchem.ncbi.nlm.nih.gov/ access May 2021), specifically from the “BioAssay Results” section.

### 2.2 Molecular modeling and molecular docking

Molecular modeling of pharmacological targets, whose structures have not already been solved, was carried out within the Schrödinger Maestro^®^ environment (Release 2021-3), specifically with the Advanced Molecular Modeling Task, as specifically explained in our previous papers (Pittalà et al., 2017; Gesualdo et al., 2021). After modeling all structures were subjected to energy minimization and optimization in implicit water model, or in an implicit water and membrane model. Covalent or non-covalent molecular docking has been carried out with Glide molecular docking task, within the Schrödinger Maestro^®^ environment. All poses (the relative conformation of ligands bound to the pharmacological target) were rescored with MM-GBSA calculations. MM-GBSA calculations provide prediction of binding free energy (ΔG_binding_) and relative energy contributions to it. Experimental protocols are the same as reported for Keap1 and melanocortin receptor hMC1R and hMC5R, by Pittalà et al. (2017); Pittalà et al. (2017); Gesualdo et al. (2021); and Gesualdo et al. (2021), respectively.

### 2.3 Molecular dynamics of melanocortin receptors

All-atom molecular dynamics simulation of hMC1R bound to axitinib, BMS-470539 (BMS, selective MC1 agonist) and agouti-Related Protein (AgRP, selective MC1 antagonist) as predicted through molecular docking was carried out inserting protein complexes tin an explicit water-membrane system, with Desmond Molecular Dynamics Simulation Task of Schrödinger Maestro (Release 2023-3). Specifically, an orthorhombic box has been created, the receptors were included in a 30 Å3 POPC lipid membrane-water system according to output from OMP database (https://opm.phar.umich.edu/). TIP3P water model has been selected molecules were added to the system, along with NaCl (150 mM). After membrane protein equilibration protocol, 20 ns NPγT ensemble production runs were carried out. Simulation Interaction task of Schrödinger Maestro provided information regarding ligand-receptor interactions. Visual Molecular Dynamics software (VMD version 1.9.3) (Humphrey et al., 1996) provided analysis of salt-bridges and contact maps of hMC1R complexes. Fuzzy Logic algorithm through access to the web server (https://online-image-comparison.com/), setting the fuzz option cut-off value to 4, was used to highlight the differences between contact maps of different complexes.

### 2.4 Cell culture and treatments

Retinal endothelial cells are the cell type which are mainly exposed to high glucose blood concentration during diabetes; thereby the cell line used in this manuscript represents the best *in vitro* model to resemble blood retinal barrier breakdown upon high glucose exposure. Primary human retinal endothelial cells (HRECs) were purchased from Innoprot^®^ (P10880; Derio–Bizkaia, Spain). Cells (passage n° 3–4) were cultured at 37°C, in humidified atmosphere (5% CO_2_), in Endothelial Cell Medium (ECM) supplemented with 5% fetal bovine serum (FBS), 1% ECGS (Endothelial Cell Growth Supplement) and 100 U/mL penicillin 100 μg/mL streptomycin, in flask precoated with fibronectin (1 mg/mL) (P60104–P8248; Innoprot, Derio–Bizkaia, Spain) for 1 h at 37°C. After reaching confluence (approximately 70%), cells were used for experimental procedures. Cells growth in medium containing 5 mM glucose (physiological glucose concentration) served as control group. We divided cell challenges and treatments in two experimental models: model A–HRECs exposed to high glucose levels (40 mM) continuously for 48 h (Lazzara et al., 2019; Lazzara et al., 2022); model B–HRECs exposed to fluctuating glucose levels for 72 h (5–40 mM glucose concentration medium shift every 12 h, within 72 h time frame) (Sun et al., 2010; Mudaliar et al., 2014; Maeda et al., 2015). In both models HRECs were treated with axitinib (0.1-1-10-25 µM, PZ0193, Sigma-Aldrich, St. Louis, MO, United States) and then were exposed to continuous or fluctuating HG.

### 2.5 MTT assay

The 3-[4,5-dimethylthiazol-2-yl]-2,5-diphenyl tetrasodium bromide (MTT; M-2128, Sigma-Aldrich, St. Louis, MO, United States) was used to assess cell viability after treatment with axitinib (0.1-1-10–25 µM) with or without HG (40 mM) challenge. Optimal cell density was obtained by seeding 1.5 × 10^4^ cells/well in 96-well plates (Costar, Corning, NY, United States). HRECs were subjected to treatment in a fresh medium for 48 h with axitinib with or without HG (40 mM). At the end of the treatment, HRECs were incubated at 37°C with MTT (5 mg/mL) for 3 h; then DMSO was added, and absorbance was measured at 570 nm in a plate reader (VariosKan, Thermo Fisher Scientific, Waltham, MA, United States). Graphs were built converting absorbance (abs) to viability (% of control) using the following equation (abs_x_ ÷ abs_ctrl−_) × 100, where abs_x_ is absorbance in the x well, and abs_ctrl−_ is the average absorbance of negative control cells (untreated cells).

### 2.6 Lactate dehydrogenase (LDH) cell release

Lactate dehydrogenase (LDH) cell release was measured using the CyQUANTTM LDH Cytotoxicity assay (C20301, ThermoFisher, Waltham, Massachusetts, United States). HRECs cells were seeded at 1,5 × 10^4^ cells/well in 96-well plates (Costar, Corning, NY, United States). HRECs were subjected to treatment in a fresh medium for 48 h with axitinib (0.1–1–10–25 µM) with or without HG (40 mM). After these time points, according to manufacturer’s protocol, lysis solution was added to positive control wells (non-treated cells) for 45 min. After transferring 50 μL of medium in a new multi-well plate, 50 μL of working solution was added. After 10–15 min at room temperature, at last, 50 μL of stop solution was added. The absorbance values were measured at 490 nm using a plate reader (VarioSkan, Thermo Fisher Scientific, Waltham, MA, United States). LDH release is normalized to control; absorbance values were corrected by subtracting medium absorbance.

### 2.7 Reactive oxygen species production

ROS were measured by a 2′,7′-dichlorofluoresceindiacetate (DCFDA) Cellular Reactive Oxygen Species Detection Assay Kit (113851, Abcam, Cambridge, United Kingdom). Optimal cell density was obtained by seeding 1.5 × 10^4^ cells/well in 96-well plates (Costar, Corning, NY, United States). After reaching confluence, HRECs were subjected to treatment in a fresh medium for 48 h with axitinib (0.1–1–10–25 µM) with or without HG (40 mM). After treatment, media were aspirated and cells were washed by adding 100 µL/well of 1X buffer, according to manufacturer’s protocol; after washing, HREC cells were stained by adding 100 µL/well of the diluted DCDFA solution (25 µM). Cells were also incubated with this solution for 45 min at 37°C in the dark. After removing DCDFA solution, 100 µL/well of 1X buffer was added and ROS concentration was measured immediately by detection of DCF fluorescence (λ_ex_ = 495 nm, λ_em_ = 529 nm) with a Varioskan™ Flash Multimode Reader. Results were reported normalizing the sample fluorescence to the fluorescent intensity of control cells (untreated cells).

### 2.8 Western blot analysis

HRECs were cultured in 60 mm Petri dishes (4 × 10^5^). Proteins of whole cell lysates were extracted with RIPA Buffer, including protease and phosphatase inhibitors cocktail (R0278; P8340; P0044; P5726, Sigma-Aldrich, St. Louis, MO, United States). Total protein content, in each cell lysate sample, was determined by means of the BCA Assay Kit (23225, Pierce™ BCA Protein Assay Kit, Invitrogen, Life Technologies, Carlsbad, United States). Extracted proteins (30 μg) were loaded on 4%–12% tris–glycine gel (NP0322, Invitrogen, Thermo Fisher Scientific, Carlsbad, CA, United States). After electrophoresis, proteins were transferred into a nitrocellulose membrane (IB23002, Invitrogen, Thermo Fisher Scientific, Carlsbad, CA, United States). Membranes were blocked with milk 5% in tris buffered saline, and 0.2% Tween 20 (TBST) for 1 h at room temperature. Membranes were incubated overnight (4°C) with appropriate primary phospho-p44/42 MAPK (Rabbit, phospho-Erk1/2, 1:500 dilution, Cat. n. 9101, Cell Signaling Technology, Danvers, MA, United States), p44/42 MAPK (Rabbit, Erk1/2, 1:500 dilution, Cat. n. 9102, Cell Signaling Technology, Danvers, MA, United States), NRF2 (1:500 dilution, Cat. n. 12721, Cell Signaling Technology, Danvers, MA, United States), phospho-VEGF Receptor 2 (1:500 dilution, Cat. n. 2478, Cell Signaling Technology, Danvers, MA, United States), phospho-VEGF Receptor 1 (1:1000 dilution, Cat. n. SAB4504006, Sigma-Aldrich St Louis, MO), GAPDH (1:1000 dilution, Cat. n. 2118S, Cell Signaling Technology, Danvers, MA, United States) and anti-beta actin (1:1000 dilution, Cat. n. A2066, Sigma-Aldrich St Louis, MO). After overnight incubation, the membranes were then incubated with secondary chemiluminescent antibodies (ECL anti-mouse, NA931 and ECL anti-rabbit, NA934, 1:2000 dilution, Cytiva Amersham, United Kingdom) for 1 h at room temperature. After secondary antibodies, the membranes were incubated with ECL (34580, SuperSignal™ West Pico PLUS Chemiluminescent Substrate, Thermo Fisher Scientific, Carlsbad, CA, United States) and were detected through I-Bright™ 1500 (Invitrogen, Life Technologies, Carlsbad, CA, United States) by using chemiluminescence. Densitometry analyses of blots were performed at non-saturating exposures and analyzed using ImageJ software (NIH, Bethesda, MD). Values were normalized to GAPDH and actin.

### 2.9 Statistical analysis

*In vitro* studies have been carried out by two researchers that were blind to group labels, a third researcher carried out statistical analysis, after that group labels were unveiled and graphs images were generated. All results are reported as mean ± SD (*n* = 5). Data were normally distributed (Shapiro-Wilk test, SPSS Crayon Group). The results were analyzed using one-way ANOVA followed by Tukey-Kramer *post hoc* multiple comparisons test, given F with a *p* < .05, and no significant variance inhomogeneity. Differences between groups were considered significant for *p*-value <0.05. Graph design and statistical analysis were carried out using GraphPad prism 7 (GraphPad software La Jolla, California).

## 3 Results

### 3.1 Covalent docking of axitinib at Keap1

**FIGURE 1:**
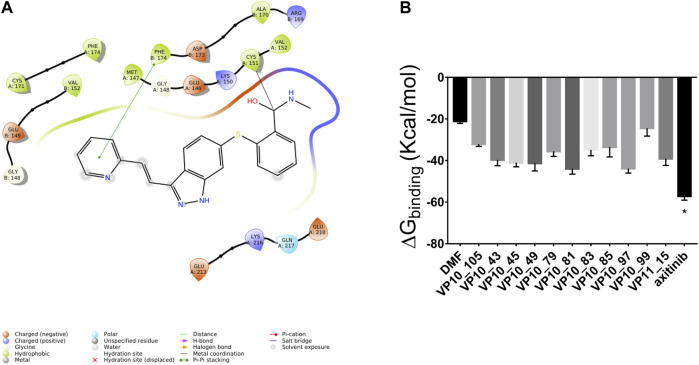
Axitinib modulates Nrf2 expression through a nucleophilic addition to Cys151 of Keap1, to amide bond of the compound. **(A)** 2D representation of axitinib covalently bound to Cys151 of Keap1. **(B)** Predicted ΔG_binding_ of known Keap1 binders (simulated nucleophilic addition to Cys151).

The covalent docking simulation predicted for axitinib a binding to Keap1 through a nucleophilic addition to the amide bond of axitinib, rather than a Michael addition. Michael addition is not a favorable reaction between axitinib and Cys151, as double bond flanked between pyridine ring and indazole ring is not sufficiently activated to act as Michael acceptor. Specifically, we found that predicted ΔG_binding_ of axitinib, for nucleophilic addition, was the lowest (more favorable) compared to other screened compounds (proprietary database), already tested *in vitro* as covalent ligands of Nrf2 (Figure 1).

### 3.2 *In silico* target-scouting

**FIGURE 2:**
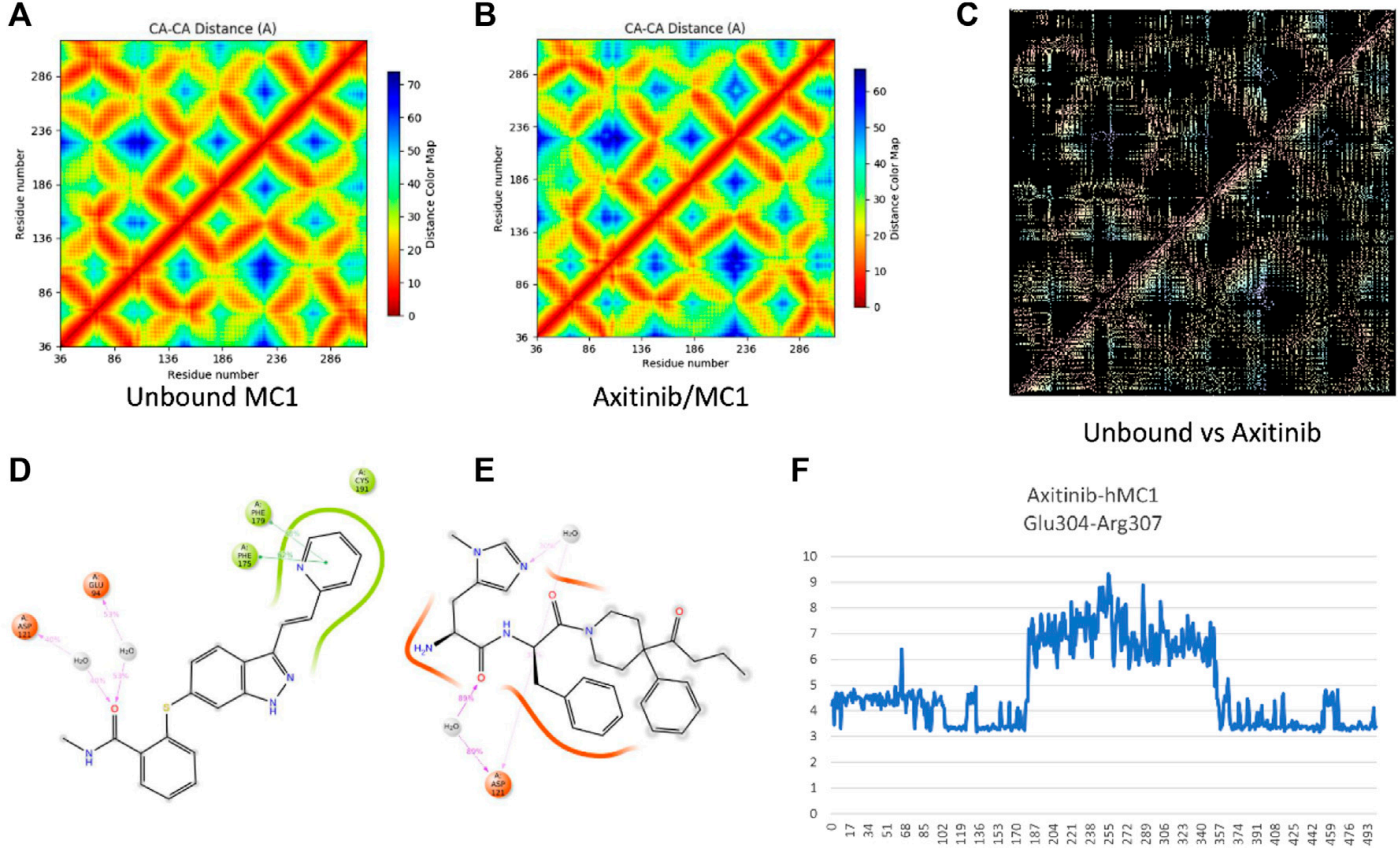
Axitinib binding to MC1R. **(A)** Residue-residue contact map for unbound MC1 receptor after 20 ns simulation. **(B)** Residue-residue contact map for axitinib/MC1 receptor complex after 20 ns simulation. **(C)** Differences in residue-residues contact maps in hMC1R upon binding with axitinib, this map is highly similar to difference-map for the BMS/MC1 receptor complex, as previously reported [17]. Panels **(D,E)** represent bidimensional interaction of axitinib **(D)** and the selective MC1R agonist BMS **(E)** during 20 ns simulations; red ribbons represent a negative charged protein surfaces, while green ribbons represent hydrophobic protein surfaces; magenta arrows represent water-bridges and green segments represent π-π stacking interactions. **(F)** Glu304-Arg307 distance (Å) over simulation frame #, for a total of 500 frames representing 20 ns of MD simulation of axitinib-hMC1 complex.

Alternative targets of axitinib have been identified through the swiss target prediction server, including some GPCRs. According to the preliminary pre-clinical studies supporting that melanocortin receptors (MC1R and MC5R) agonists exert anti-angiogenic activity in *in vitro* and *in vivo* models of diabetic retinopathy, we planned to explore the binding of axitinib to these receptors through a molecular modeling approach, molecular dynamics and docking simulations, as previously described by Gesualdo et al. in 2021 (Gesualdo et al., 2021). Thereafter, axitinib has been docked along with other known MC1 and MC5 receptor ligands, and docking has been rescored through MM-GBSA calculations. Pose-clustering option suggested that axitinib would bind preferentially to hMC1R. We were not able to identify for axitinib any pose or cluster as putative selective ligand for MC5 receptor. The complexes BMS-470539/hMC1R and axitinib/hMC1R were then subjected to MM-GBSA rescoring. BMS has shown a slightly more favorable binding energy (−85 kcal/mol) to hMC1R, compared to axitinib (−62 kcal/mol). Therefore, we carried out molecular dynamics of axitinib/hMC1R and compared results to simulation previously carried out for BMS/hMC1R and AgRP/hMC1R complexes (Gesualdo et al., 2021), to predict if axitinib binds as agonist or antagonist of MC1R. Looking at differences between residue-residue contact maps of unbound MC1 and agonist/antagonist-hMC1 complex (Figures 2A,B), we analyzed the conformational modification of receptor upon binding. Specifically, the difference pattern in the contact-map of hMC1R induced by axitinib (Figure 2C), resembled the pattern induced by the selective MC1R agonist BMS, accordingly to data previously shown for fingolimod (Gesualdo et al., 2021). The molecular dynamics simulation (20 ns) evidenced that axitinib binds to MC1 receptor mainly through π-π stacking hydrophobic interactions with Phe 175 (64% frequency) and Phe 179 (38% frequency); while hydrophilic interactions occur through water-bridges with Asp 121 and Glu 94, with about 40% of frequency (Figure 2D). On the contrary, BMS (Figure 2E) mainly interacts with MC1 receptor through water-bridges including Asp 121, by means of 80% frequency for 20 s simulations. These differences may explain the slightly higher affinity of BMS for hMC1R compared to axitinib. Salt-bridge analysis of simulations confirmed that axitinib induced in hMC1R a conformational modification similar to the agonist BMS. Specifically, axitinib stabilized during 2 thirds of 20 ns simulation a salt-bridge (Glu304-Arg307, 4 Å distance) (Figure 2F), which was destroyed in the AgRP-hMC1R complex as reported by Gesualdo et al., 2021; Gesualdo et al., 2021).

**FIGURE 3:**
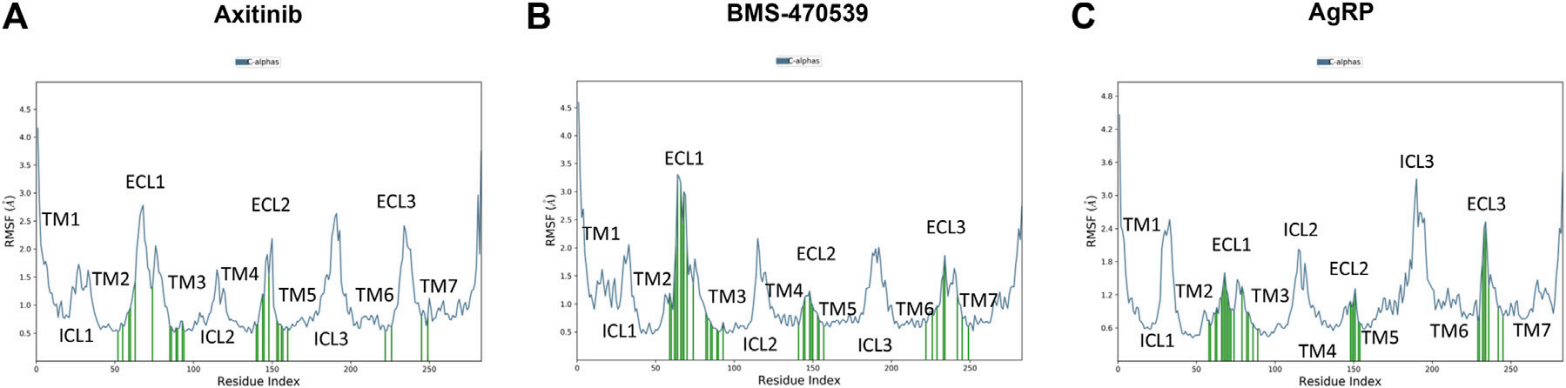
Root mean square fluctuation of analyzed complex and ligand receptor contacts. **(A)** RMSF in Angstrom Å (blu line) of axitinib-hMC1 complex, green lines represent the contacts between axitinib and the receptor. **(B)** RMSF in Angstrom Å (blu line) of BMS-hMC1 complex, green lines represents the contacts between BMS and the receptor. **(C)** RMSF in Angstrom Å (blu line) of AgRP-hMC1 complex, green lines represents the contacts between AgRP and the receptor. ICL stands for intracellular loop, ECL stands for extracellular loop and TM stands for transmembrane helices.

Axitinib, BMS and AgRP interacts with the same regions of hMC1 receptor (Figure 3). Besides higher number of contacts of BMS with helices 2 and 3 and the extra cellular loop 1 (ECL1), the ECL1 in the BMS-hMC1 complex has a greater flexibility (3.5 Å root mean square fluctuaction, RMSF), compared to axitinib-hMC1 complex (2.7 Å RMSF) (Figures 3A,B). AgRP (Figure 3C) decreased to 1.2 Å RMSF of ECL1, in comparison to ECL1 RMSF in the axitinib (3 Å RMSF) and BMS complexes (3.5 Å RMSF) and increased consistently the RMSF of the first intracellular loop (ICL1) and of the third intracellular and extracellular loops (ICL3 and ECL3) of hMC1. Thereby, the MC1 antagonist AgRP increased RMSF of melanocortin receptor 1, with exception to ECL1.

### 3.3 Effects of axitinib in primary retinal endothelial cells exposed to high glucose

**FIGURE 4:**
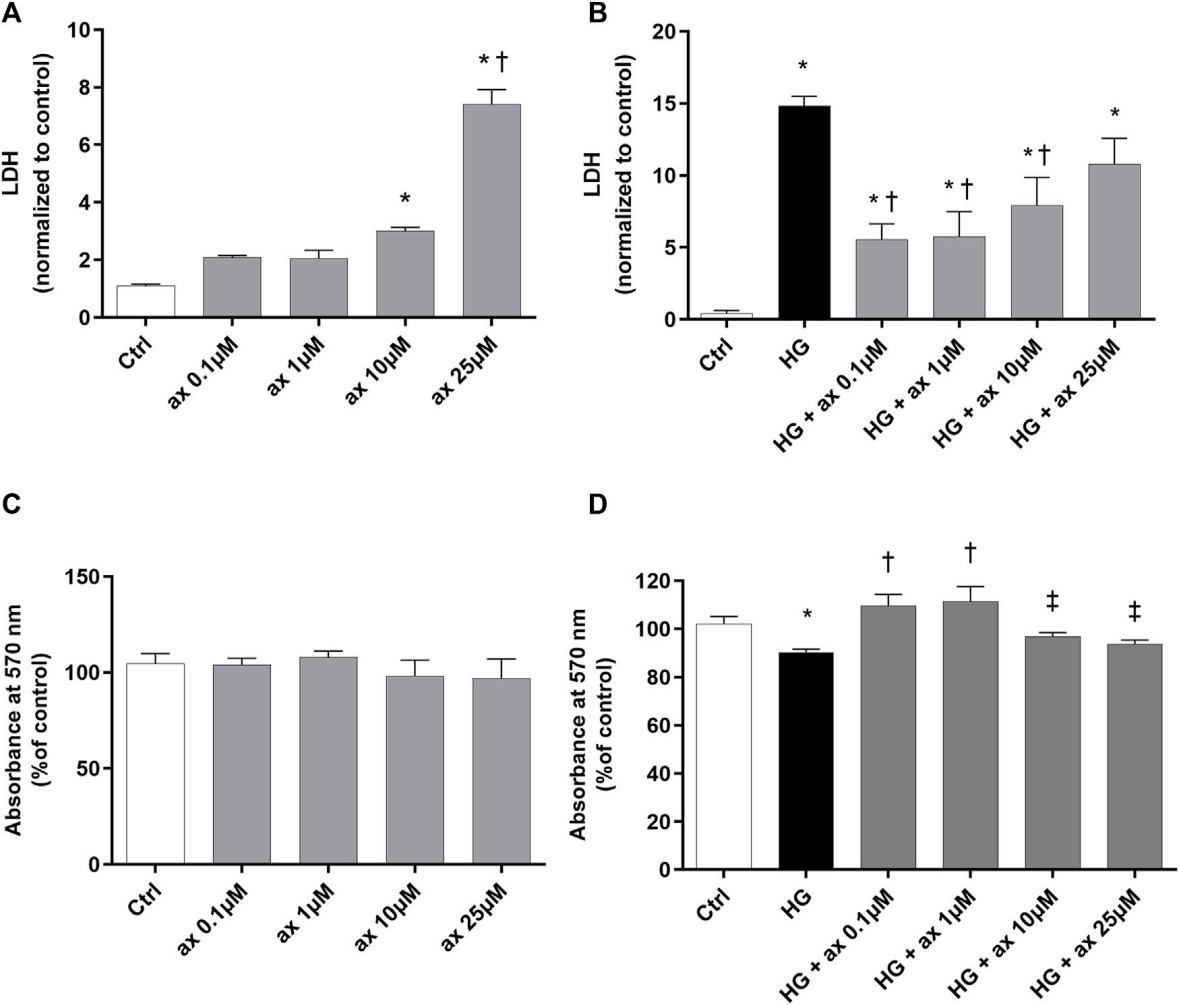
Axitinib reduced cytotoxicity in HRECs growth in high glucose. Axitinib was well tolerated by primary human retinal endothelial cells up to 10 µM. LDH and MTT assays were carried out on HRECs treated with axitinib (ax; 0.1, 1, 10, 25 µM) in normal glucose-medium **(A,C)** and in high glucose medium (HG) **(B,D)**. Mean value of control photometric absorption at λ 570 nm = 0.128. Values are reported as mean ± SD; *n* = 5. Data were analyzed by one-way ANOVA and the Tukey *post hoc* test for multiple comparisons. *p < 0.05 *vs*. control; ^†^ *p* < 0.05 *vs.* ax 0.1, 1, 10 µM **(A)** and *vs.* HG **(B,D)**; ^‡^ *p* < 0.05 *vs.* ax 0.1 µM and 1 µM **(D)**.

HRECs have been exposed to different concentrations of axitinib (0.1–25 µM), at normal levels of glucose (control - ctrl, 5 mM) and at high levels of glucose (HG, 40 mM) for 48 h. HRECs tolerability to axitinib has been evaluated through LDH (Figures 4A,B) and MTT (Figures 4C,D) assays. The 25 µM axitinib concentration has been found to be toxic to HRECs, leading to significant LDH release compared to control cells (Figure 4A). High glucose challenge induced cell toxicity after 48 h (Figure 4B) and axitinib was able to protect endothelial cells, with a significant (*p* < 0.05) reduction of LDH release (Figure 4B). Furthermore, all tested concentrations of axitinib did not significantly affect cell viability of HRECs growth in 5 mM glucose medium (Figure 4C). However, only axitinib 0.1 and 1 µM significantly (*p* < 0.05) increased HRECs cell viability, compared to HRECs exposed for 48 h to high glucose levels (MTT, Figure 4D). For this reason, we used 0.1 and 1 µM axitinib for all subsequent experiments.

**FIGURE 5:**
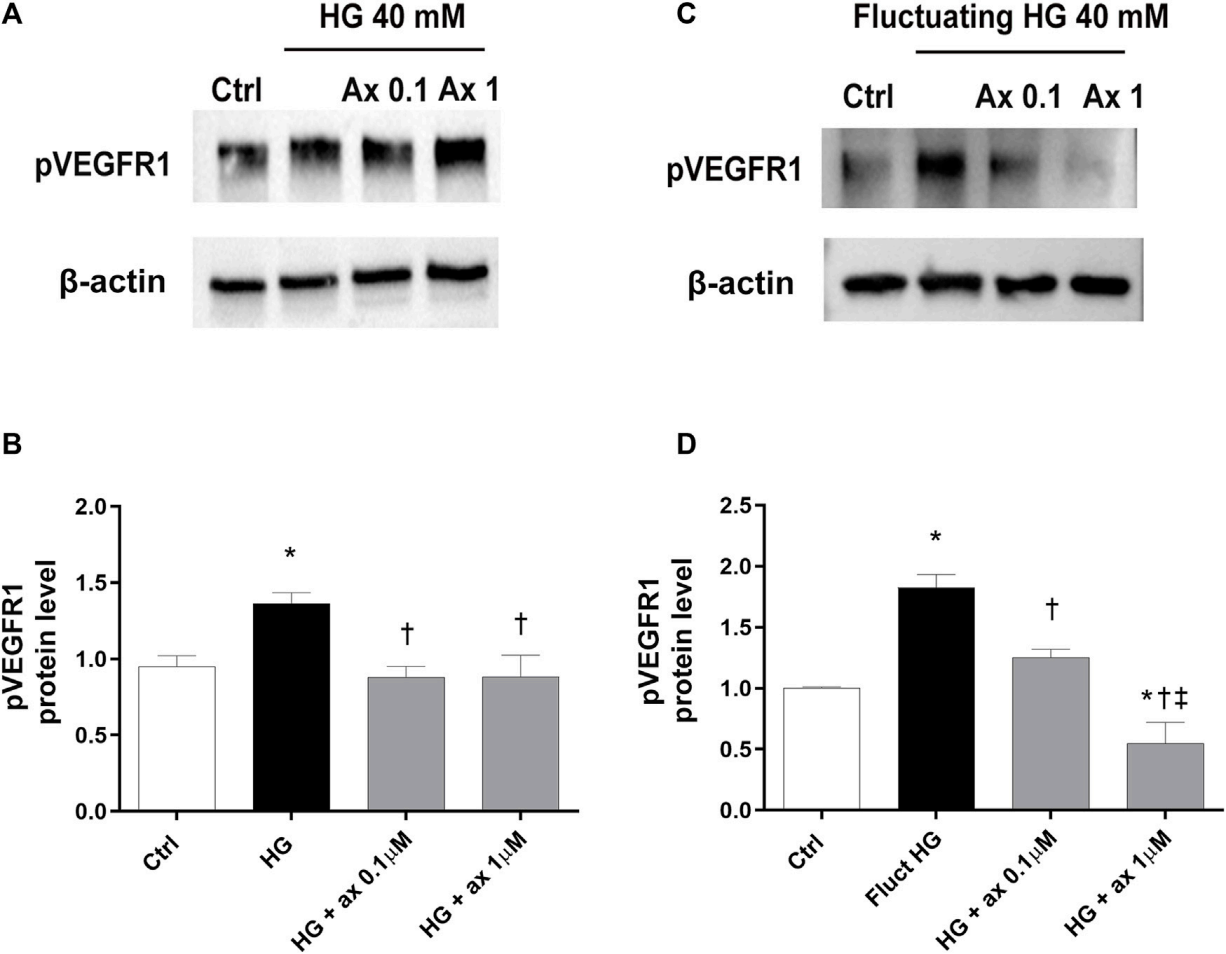
Axitinib counteracted VEGFR1 activation in HRECs in two *in vitro* models of DR. Western blot was carried out on HRECs treated with axitinib (ax, 0.1, 1 µM) and high glucose (HG). **(A,B)** Immunoblot analysis and densitometric analysis of phosphorylated VEGFR1 in lysates of HRECs treated with axitinib and HG for 48 h (model A). **(C,D)** Immunoblot analysis and densitometric analysis of phosphorylated VEGFR1 in lysates of HRECs treated with axitinib and fluctuating (fluct) HG for 72 h (model B). Densitometry analysis of each band was carried out with the ImageJ program, VEGFR1 phosphorylation has been normalized to β-actin values. Values are reported as mean ± SD; *n* = 5. Data were analyzed by one-way ANOVA and the Tukey *post hoc* test for multiple comparisons. *p < 0.05 *vs.* control, ^†^ *p* < 0.05 *vs.* HG; ^‡^ *p* < 0.05 *vs.* ax 0.1 µM.

We explored the activation of VEGFR1 and VEGFR2 receptors in our *in vitro* models of DR, through Western blot analysis of phosphorylated receptors. In HRECs exposed to 40 mM glucose concentration for 48 h (model A; Figures 5A,B) and in cells exposed for 72 h to fluctuating glucose concentration (model B, Figures 5C,D) we found significantly (*p* < 0.05) increased levels of phosphorylated VEGFR1, compared to control cells (Figure 5). As expected, the treatment with axitinib (0.1 and 1 µM) significantly (*p* < 0.05) reduced the phosphorylation of VEGFR1 in both tested *in vitro* models of DR (model A, Figures 5A,B and model B Figures 5C,D).

**FIGURE 6:**
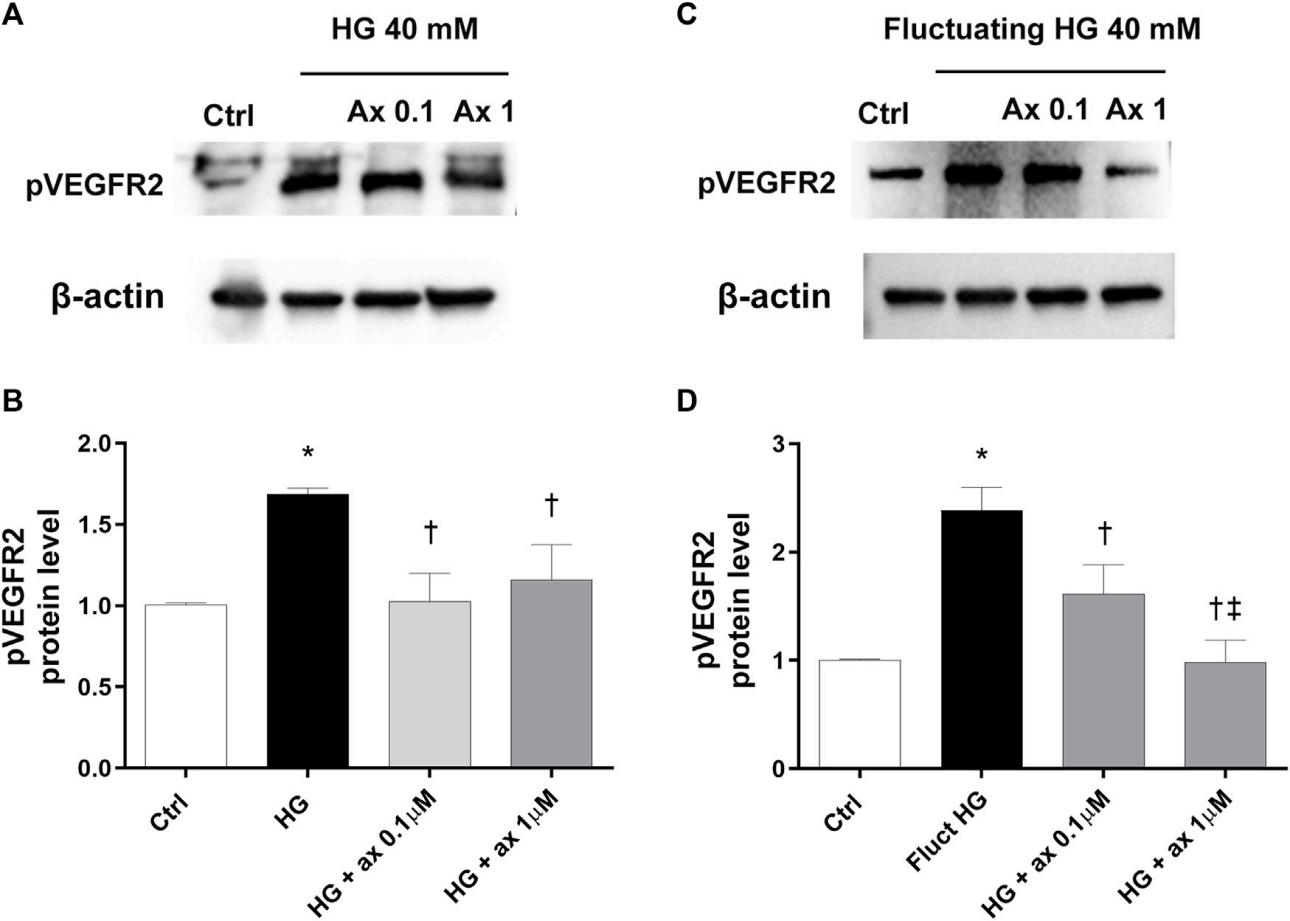
Axitinib decreased VEGFR2 phosphorylation in HRECs in two *in vitro* models of DR. Western blot was carried out on HRECs treated with axitinib (ax; 0.1, 1 µM) and high glucose (HG). **(A,B)** Immunoblot analysis and densitometric analysis of phosphorylated VEGFR2 in lysates of HRECs treated with axitinib and HG for 48 h (model A). **(C,D)** Immunoblot analysis and densitometric analysis of phosphorylated VEGFR2 in lysates of HRECs treated with axitinib and fluctuating (fluct) HG for 72 h (model B). Densitometry analysis of each band was carried out with the ImageJ program, VEGFR2 phosphorylation has been normalized to β-actin values. Values are reported as mean ± SD; *n* = 5. Data were analyzed by one-way ANOVA and the Tukey *post hoc* test for multiple comparisons. *p < 0.05 *vs.* control; ^†^ *p* < 0.05 *vs.* HG; ^‡^ *p* < 0.05 *vs.* ax 0.1 µM.

Increased expression of phosphorylated VEGFR2 (pVEGFR2) has been found in HRECs exposed to high glucose concentration in both models of DR (model A and B) (Figure 6). Cell treatment with axitinib (0.1, 1 µM) significantly (*p* < 0.05) reduced the phosphorylation of VEGFR2 in HRECs exposed to continuous high glucose concentration (model A; Figures 6A,B) and also to fluctuating glucose concentration (model B; Figures 6C,D).

**FIGURE 7:**
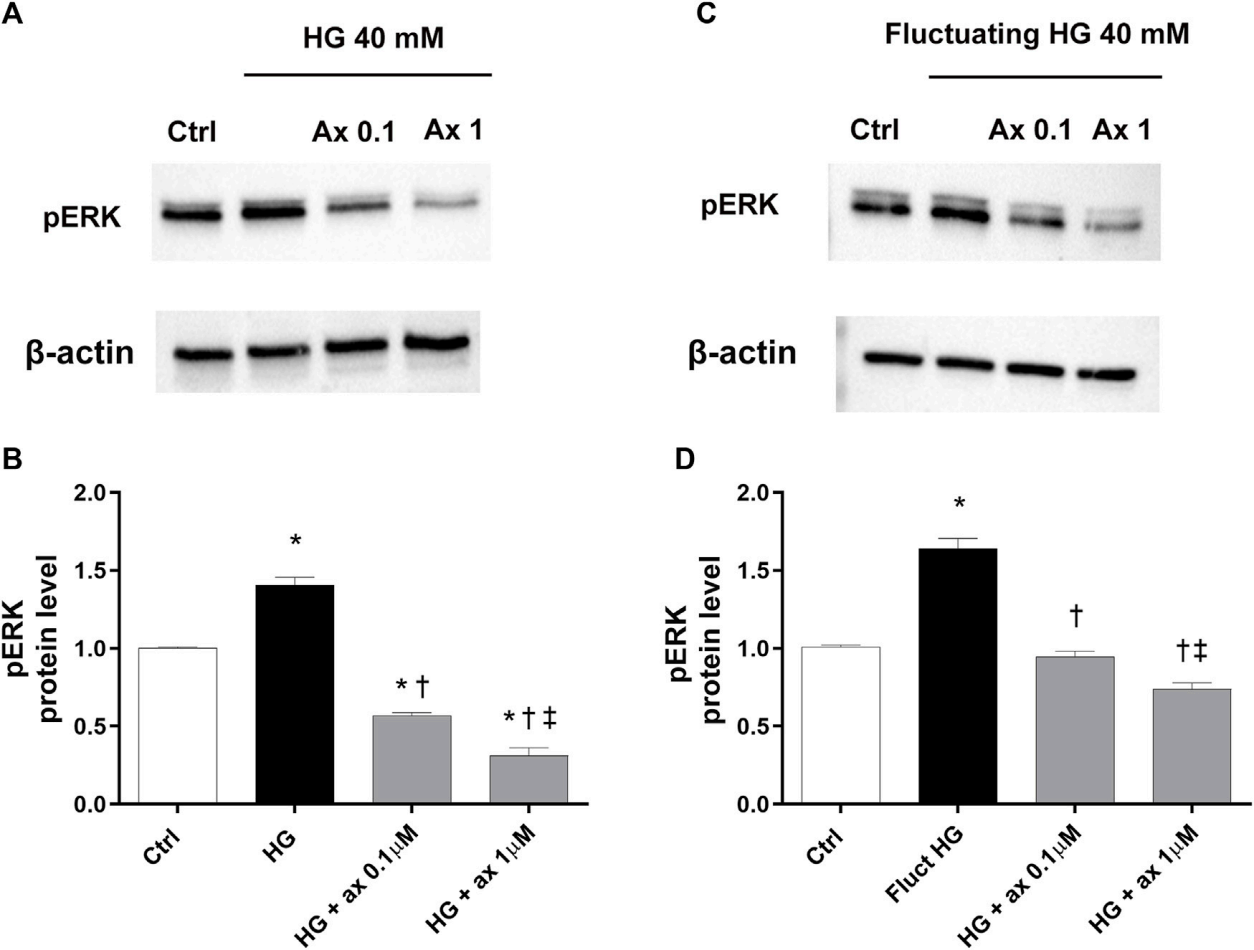
Axitinib reduced pERK pathway activation in HRECs in two *in vitro* models of DR. Western blot was carried out on HRECs treated with axitinib (ax; 0.1, 1 µM) and high glucose (HG). **(A,B)** Immunoblot analysis and densitometric analysis of phosphorylated ERK in lysates of HRECs treated with axitinib and HG for 48 h (model A). **(C,D)** Immunoblot analysis and densitometric analysis of phosphorylated ERK in lysates of HRECs treated with axitinib and fluctuating (fluct) HG for 72 h (model B). Densitometry analysis of each band was carried out with the ImageJ program, ERK phosphorylation has been normalized to beta-actin values. Values are reported as mean ± SD; *n* = 5. Data were analyzed by one-way ANOVA and the Tukey *post hoc* test for multiple comparisons. *p < 0.05 *vs.* control; ^†^ *p* < 0.05 *vs.* HG; ^‡^ *p* < 0.05 *vs.* ax 0.1 µM.

According to the well-established mechanism of action of axitinib, we also analyzed the activation of the ERK pathway in both two *in vitro* models of DR. We found significantly (*p* < 0.05) increased phosphorylation of ERK protein in HRECs exposed to HG for 48 h and to fluctuating glucose concentrations, compared to control cells (Figure 7). Treatment with axitinib (0.1, 1 µM) significantly reduced levels of phosphorylated ERK protein, compared to cells exposed to continuous HG levels (model A; Figures 7A,B) and to fluctuating glucose concentrations (model B; Figures 7C,D).

**FIGURE 8:**
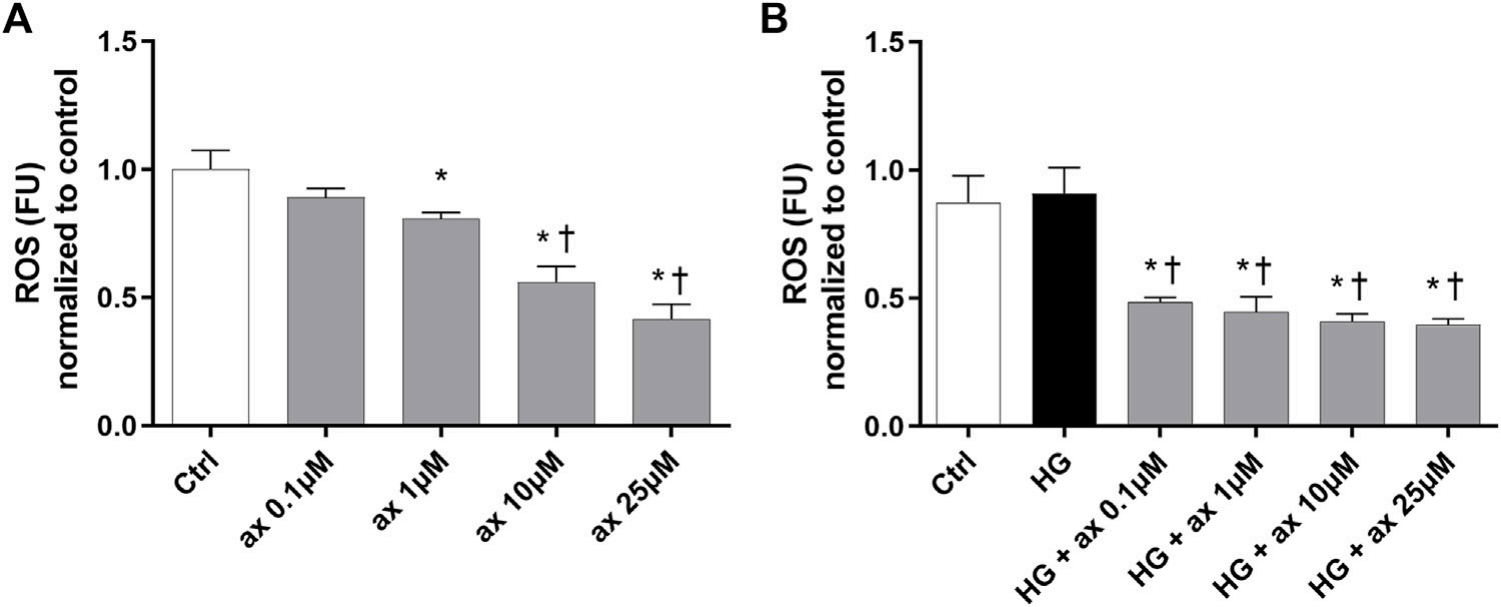
Axitinib exerted antioxidant activity in HRECs. DCFDA assay was carried out on HRECs treated with axitinib (ax; 0.1, 1, 10, 25 µM) in normal glucose medium **(A)** and in high glucose (HG) medium **(B)** for 48 h. ROS, reactive oxygen species; FU, Fluorescence unit. Values are reported as mean ± SD; *n* = 5. Data were analyzed by one-way ANOVA and the Tukey *post hoc* test for multiple comparisons. *p < 0.05 *vs.* control, ^†^ *p* < 0.05 *vs.* ax 0.1, 1 µM **(A)**, *vs.* HG **(B)**.

We determined the levels of ROS in HRECs, growth in 5 mM glucose medium (ctrl) and treated with different concentrations of axitinib (0.1–25 µM). Axitinib, at 1–25 µM concentration range, significantly decreased ROS production in HRECs, in a concentration-response manner (Figure 8A), then exerting an antioxidant action. Similarly, at all tested concentrations (0.1–25 µM), axitinib significantly decreased ROS in HRECs exposed for 48 h to high glucose levels (Figure 8B).

**FIGURE 9:**
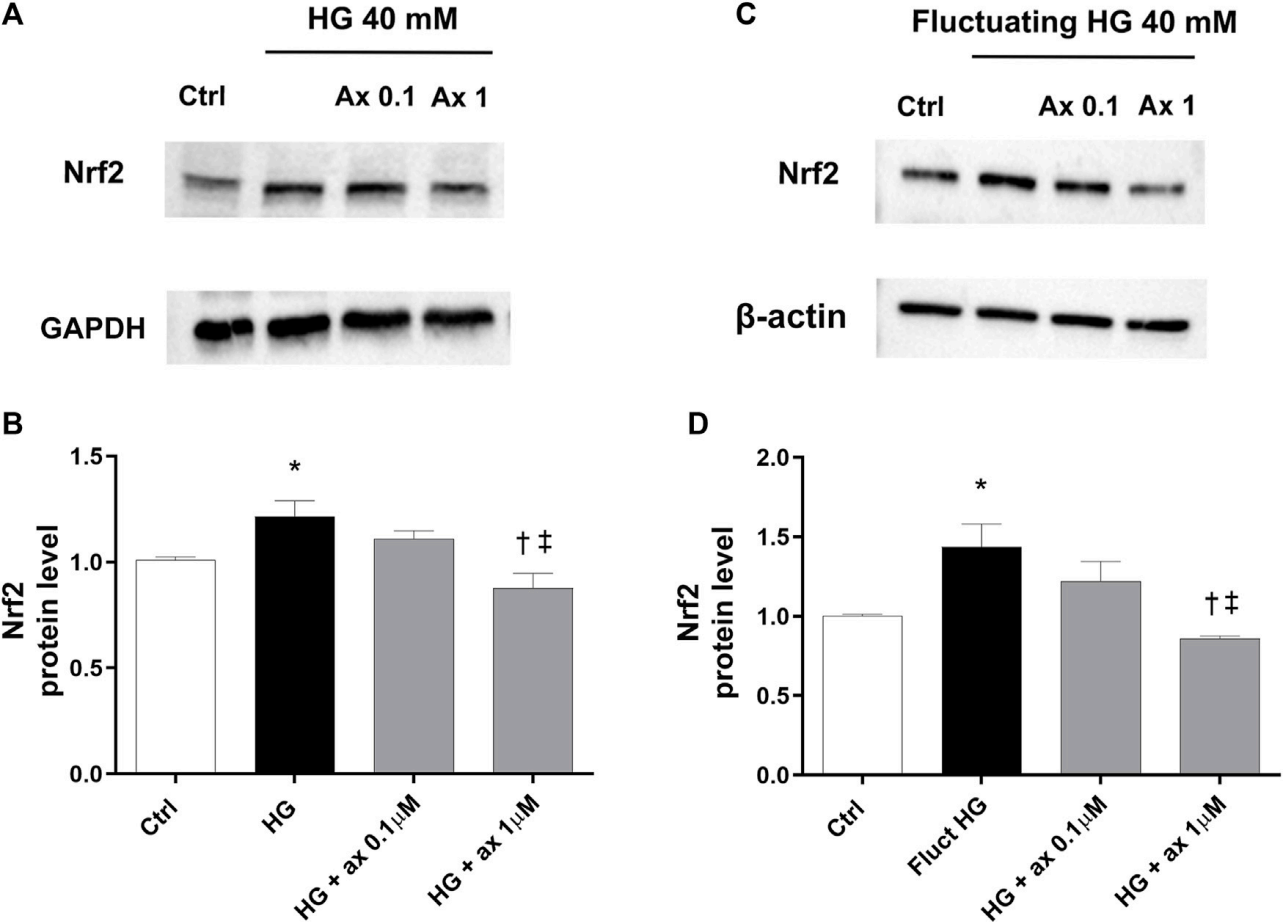
Axitinib reduced Nrf2 expression in HRECs in two *in vitro* models of DR. Western blot was carried out on HRECs treated with axitinib (ax; 0.1, 1 µM) and high glucose (HG). **(A,B)** Immunoblot analysis and densitometric analysis of Nrf2 in lysates of HRECs treated with axitinib and HG for 48 h (model A). **(C,D)** Immunoblot analysis and densitometric analysis of Nrf2 in lysates of HRECs treated with axitinib and fluctuating (fluct) HG for 72 h (model B). Densitometry analysis of each band was carried out with the ImageJ program, Nrf2 expression has been normalized to GAPDH and β-actin values. Values are reported as mean ± SD; *n* = 5. Data were analyzed by one-way ANOVA and the Tukey *post hoc* test for multiple comparisons. *p < 0.05 *vs.* control, ^†^ *p* < 0.05 *vs.* HG; ^‡^ *p* < 0.05 *vs.* ax 0.1 µM.

Accordingly, we further explored the expression of the Nrf2 protein in two *in vitro* models of DR (48 h exposure to 40 mM glucose concentration–model A; 72 h of fluctuating glucose concentration–model B), to ascribe to the antioxidant activity of axitinib the involvement of Nrf2. In both models HG-treated HRECs expressed significant (*p* < 0.05) higher levels of Nrf2 compared to control cells (5 mM medium), as shown in Figures 9A,B (model A) and Figures 9C,D (model B). Axitinib (0.1 and 1 µM) significantly (*p* < 0.05) reduced Nrf2 expression to control levels.

## 4 Discussion

Axitinib is currently indicated for the treatment of RCC, wherever the first line treatment with sunitinib fails. Axitinib IC_50_ for VEGFR1 and VEGFR2 is about 2 logs lower than sunitinib. Particularly, sunitinib administration has been associated with ocular complications (Fraunfelder and Fraunfelder, 2018; Santana-Garrido et al., 2020). On the contrary, with exception of two case studies (Jenkins et al., 2021; Pyare et al., 2023), no ocular adverse events have been observed for axitinib. Moreover, axitinib and sunitinib ocular adverse events were correlated to the dose (Dib et al., 2012; Jenkins et al., 2021). Thereby, compared to sunitinib, axitinib would be more suitable for pharmaceutical development of an ocular topical formulation for treatment of retinal proliferative diseases, also based on its established pharmacodynamic profile as potent VEGFR inhibitor, although several chemical-physical properties of axitinib make the design of eye drops formulation challenging (Jansook et al., 2024).

Besides its validated activity as tyrosine kinase receptor inhibitor, we found in our *in vitro* models of diabetic retinopathy that axitinib exerts antioxidant, anti-inflammatory and anti-angiogenic effects, also regulating Nrf2 expression, likely through covalent binding to Keap1-BTB domain. Axitinib is an electrophilic compound which inhibits VEGFR tyrosine kinase domain, through covalent bonds to cysteine residues, this reaction occurs through nucleophilic addition (Gotink and Verheul, 2010; Zhao and Bourne, 2018). We found that axitinib was able to react through nucleophilic addition with a cysteine residue of Keap1, which is a sensor of oxidative stress, promoting induction of Nrf2 and of cell defense machinery to oxidative stress. This prediction is supported also by a study reporting the ability of axitinib to promote Nrf2 expression (Huang et al., 2019) in *in vitro* models of RCC. Furthermore, the downstream proteins of Nrf2, NQO1 and HO-1 were significantly enhanced in renal cancer cells under treatment with axitinib (Huang et al., 2019). On the contrary, sunitinib was found to be a negative regulator of Nrf2 expression (Guo et al., 2021) and this data would explain the studies reporting its toxic effects in ocular tissues, likely inducing oxidative stress (Santana-Garrido et al., 2020). The activity of axitinib through interaction with the antioxidant cell machinery, specifically through Nrf2, was proven in our *in vitro* models of DR. Specifically, the Nrf2-antioxidant system works through an hormetic response (Calabrese and Kozumbo, 2021). Nrf2 can be activated by several stressors, including high glucose stimuli (Wang et al., 2020), and if overactivated would negatively impact cell response.

The standard therapies for advanced stage of diabetic retinopathy (proliferative form, PDR) are represented by anti-VEGF therapy (aflibercept, ranibizumab) but a percentage of patients do not improve or respond to this therapy (Wallsh and Gallemore, 2021; Gurung et al., 2023). Considering that the etiopathogenesis of DR is the outcome of several detrimental activated pathways, basic research is always searching for innovative therapies to counteract the progression of the disease. In line with this, we hereby demonstrated the protective effect of axitinib on angiogenic and oxidative stress pathways activated by high glucose concentration, continuous or fluctuating. As already well reported, axitinib, as a VEGFRs inhibitor, limits cellular growth because of its anti-angiogenic properties, by directly acting on VEGFR1 and VEGFR2. Both receptors are involved in the early stages of DR, considering their activation induced by VEGF proteins family members, like VEGF-A and PlGF (Nguyen et al., 2018; Uemura et al., 2021). The activity on those receptors is essential in order to slow down the detrimental process of uncontrolled angiogenesis and inflammation, that is also linked to the activation of the cytoplasmic ERK pathway (Simó et al., 2014; Lazzara et al., 2019; Uemura et al., 2021).The angiogenic factors bind VEGFRs and lead to the phosphorylation of ERK protein with the subsequent exacerbation of angiogenesis and inflammation in DR, as already reported in retinal endothelial cells (Giurdanella et al., 2017; Lazzara et al., 2019) and in other experimental models (Miyamoto et al., 2007; Narasimhan et al., 2009; Almalki and Agrawal, 2017; Song and Finley, 2020). Axitinib, in our *in vitro* model, was able to reduce the activation of both VEGFR1 and VEGFR2, through their reduced phosphorylation and was able also to reduce the activation of ERK pathway. Thereby, axitinib exerted a significant protective effect on retinal endothelial cells against a strong challenge represented by high glucose (continuous or fluctuating exposition). Giddabasappa et al., demonstrated that axitinib treatment inhibits both retinal endothelial and pericyte proliferation, by blocking VEGF receptors and platelet derived growth factor (PDGF) receptors, actively involved in neovascularization (Giddabasappa et al., 2016). The same results have been reported in an *in vitro* model of diabetic macular edema, where cells were challenged with hypoxic stimuli. The authors demonstrated the significant reduction of growth factors released after axitinib treatment and the significant reduction of VEGFR1, VEGFR2 and of PDGFRb activation in RPE and HUVEC cells. Moreover, in this *in vitro* model, junction proteins were significantly overexpressed after treatment with axitinib, both in endothelial and epithelial cells (Kernt et al., 2012). Looking at current literature, we hypothesized and demonstrated for the first time, the effect of axitinib on ERK pathway in retinal endothelial cells exposed to high glucose treatment. Moreover, we have not retrieved preclinical findings about the inhibitory activity of axitinib on Nrf2 activation in retinal endothelial cells, challenged with high glucose or fluctuating glucose concentrations.

Furthermore, in the present study we explored through an already published modeling approach (Gesualdo et al., 2021) the hypothesis that axitinib would exert its protective effects on endothelial cells, exposed to high glucose levels, through binding with MC1 receptors. Several reports suggest that MC1 agonists have anti-angiogenic, anti-inflammatory and antioxidant activities (Rossi et al., 2016; Maisto et al., 2017; Can et al., 2020; Gesualdo et al., 2021; Cai et al., 2022; Garrido-Mesa et al., 2022; Ng et al., 2022; Ng and Taylor, 2023). We found through molecular docking, MM-GBSA calculation and specifically molecular dynamics simulation, that axitinib can bind to MC1 receptor as an agonist, given that the predicted axitinib/MC1 complex shared several conformational features (salt-bridges and contact maps) with the selective agonist BMS-470539-MC1 complex and not with the selective antagonists AgRP/MC1 complex. The predicted affinity of axitinib for MC1 receptor is lower compared to BMS-470539, thereby axitinib main activity is ascribed as selective tyrosine kinase inhibitor, and its predicted activity as MC1 agonist and Keap1 modulator would be accessory, promoting anti-oxidant and anti-inflammatory effects; bearing a polypharmacological profile that can be therapeutically relevant (Hopkins, 2008).

In conclusion we demonstrated that axitinib inhibits VEGFR1/VEGFR2/ERK pathways in retinal endothelial cells exposed to high glucose. We also found that axitinib is able to modulate Nrf2 expression then exerting antioxidant activity in both *in vitro* models (48 h high glucose concentrations, 72 h fluctuating glucose concentrations). Our molecular modeling studies showed that axitinib ancillary antioxidant and anti-inflammatory effects, would be linked to its activity as modulator of Nrf2 activity and MC1 agonist. This pharmacological profile suggests that axitinib could be a valid candidate to handle DR. Further studies are needed to confirm the ability of axitinib to preserve blood retinal barrier integrity, and particularly technological efforts should be carried out to develop axitinib ocular topical formulations able to deliver the drug to the retina.

## Data Availability

The original contributions presented in the study are included in the article/supplementary material, further inquiries can be directed to the corresponding author.
